# Social prescribing of nature therapy for adults with mental illness living in the community: A scoping review of peer-reviewed international evidence

**DOI:** 10.3389/fpsyg.2022.1041675

**Published:** 2022-12-06

**Authors:** Tamsin Thomas, Christina Aggar, James Baker, Debbie Massey, Megan Thomas, Daniel D’Appio, Eric Brymer

**Affiliations:** ^1^Faculty of Health, Southern Cross University, Bilinga, QLD, Australia; ^2^Northern NSW Local Health District, Lismore, NSW, Australia; ^3^Primary and Community Care Services, Thornleigh, NSW, Australia; ^4^Graduate School of Medicine, University of Wollongong, Keiraville, NSW, Australia; ^5^Manna Institute, University of New England, Armidale, NSW, Australia

**Keywords:** social prescribing, community referral, community health services, mental illness, nature therapy, ecotherapy

## Abstract

Social prescribing of nature therapy “green social prescribing” facilitates access to local nature-based activities that improve biopsychosocial wellbeing outcomes, are affordable, accessible, and can be adapted to context. These are becoming increasingly popular and gray literature is emerging, however, peer-reviewed scientific evidence is exiguous. This scoping review aimed to identify and critique peer-reviewed evidence for green social prescribing interventions and develop recommendations for research and clinical practice. Included studies were published in peer-reviewed journals in English on/after 1 January 2000. Participants were community-living adults with mental illness; Intervention was any green social prescribing program; Comparator was not restricted/required; Outcomes were any biopsychosocial measures; and any/all Study Designs were included. Twelve databases were searched on 15 October 2022; these were Academic Search Premier, APA PsycArticles, APA PsycINFO, CINAHL, Cochrane Library, Google Scholar, JSTOR, ProQuest, PubMed, Science Direct, Scopus, and Web of Science. The Mixed Methods Appraisal Tool was used to assess quality. Seven publications describing 6 unique studies (5 UK, 1 Australia) were identified including 3 mixed-methods, 2 qualitative, and 1 RCT. Participants included 334 adults (45% female, aged 35–70 years); sample sizes ranged from 9 to 164. All studies showed improvements in biopsychosocial wellbeing, and participants from most studies (*n* = 5) reported increased connection to the earth and intention to further access nature. Participant demographics and diagnoses were poorly reported, and intervention activities and assessments varied considerably. However, MMAT scores were good overall suggesting these studies may reliably demonstrate intervention outcomes. We conclude that socially prescribed nature therapy can improve biopsychosocial wellbeing and is a potentially important intervention for mental illness. Recommendations for research and clinical practice are provided.

## Introduction

The global prevalence of mental illness (MI) is estimated at 15–20%, accounting for 7–13% of disability-adjusted life-years (Vigo et al., 2016; Rehm and Shield, 2019). In industrialized countries MI costs approximately 4% of GDP (Organisation for Economic Cooperation and Development [OECD], 2021) and strains health resources (Allen, 2020). In developing countries MI is often untreated due to limited healthcare resources and infrastructure (World Health Organisation [WHO], 2021). To address this global “public health crisis” (Rosenberg, 2012), WHO prioritizes affordable community-based MI interventions that are evidence-based and address the interconnected biopsychosocial factors implicated in MI (World Health Organisation [WHO], 2004, 2021).

Social prescribing (SP) is a model of healthcare provision aligned with the WHO priorities, whereby community-based health and welfare workers refer clients to local evidence-based sources of non-medical support that address biopsychosocial wellbeing (Knapp et al., 2013; Husk et al., 2019). SP can facilitate access to therapeutic and social interventions without the need for formal healthcare services. Linkages are generally made to local existing public, private, volunteer, and faith-based services, thereby making it affordable and scalable even in countries with overwhelmed or non-existent healthcare services (Knapp et al., 2013). Examples of prescriptible programs include Alcoholics Anonymous and Park Run which span 180 and 22 developed and developing countries respectively (Alcoholics Anonymous, 2021; Parkrun Global, 2021).

Nature therapy (NT) is an umbrella term for nature-based activities designed to improve participant health and wellbeing (Shanahan et al., 2019). NT interventions are varied and include, for example, Horticulture Therapy i.e., gardening (Adevi and Mårtensson, 2013); Conservation activities e.g., tree planting (Husk et al., 2016); remote-area Wilderness Therapy including bush-skills (Rutko and Gillespie, 2013), and Forest Therapy which is a mindfulness and relaxation-based NT program (Kotte et al., 2019). Many NT programs do not require trained facilitators or specialist equipment and can be conducted in any biome. This flexibility means NT is affordable, scalable, and easily adapted to community, cultural, and environmental contexts (Burls, 2007).

In recent years there has been an increase in interest and large-scale investment in SP of NT interventions [hereafter “Green Social Prescribing” (GSP)] (Tierney et al., 2020; Lindsay et al., 2022). However, despite considerable gray literature supporting continued implementation, in particular from the UK National Health Service (National Health Service [NHS], 2019), little reliable, peer-reviewed evidence is available about how effective these interventions are for people with MI.

This paper systematically examines peer-reviewed evidence related to the biopsychosocial benefits of GSP for community-living adults with MI in terms of intervention design and research methods in order to optimize future GSP interventions and direct future research. This resource is intended to (1) inform decision-making by providing governments, non-government organizations, and other interested groups with an outline of possible interventions, the potential health outcomes, and the target beneficiaries, and (2) inform research by explicating the most important features that need to be targeted and evaluated.

There is a growing evidence base for the biopsychosocial impacts of SP (for reviews see Chatterjee et al., 2018; Leavell et al., 2019). SP as a referral pathway for MI services in general, and NT in particular, can improve access to therapeutic interventions for people with MI where formal health service are not available, thereby overcoming personal and structural barriers (Zambas and Wright, 2016; Morris et al., 2022). Referrals can come from trusted community members at a grassroots level thereby facilitating access for groups who generally have poorer mental and physical health, and may not have the skills, capacity, or resources to access formal health services; this includes socioeconomically disadvantaged populations (Allen et al., 2014; Husk et al., 2019), individuals with a disability (Lindsay et al., 2022), minority ethnic groups with cultural, religious, and language barriers (Zeh et al., 2014; Gupta, 2021), and a range of other barriers, for example in the case of Australia First Nations peoples who often do not trust formal healthcare services (Zambas and Wright, 2016).

Research has demonstrated that NT experiences can facilitate a plethora of biopsychosocial improvements. For example, NT has demonstrated improvements in physical health including the biological correlates of stress (salivary cortisol, immunoglobulin), cardiac, respiratory, and immune function (Oh et al., 2017), hypertension, obesity, post-surgical recovery, and pain (Chaudhury and Banerjee, 2020). NT also improves psychological wellbeing including improved affect and vitality, and decreased stress, depression, anxiety, and anger (Hartig et al., 1991; Ulrich et al., 1991; Lee et al., 2009; Ryan et al., 2010; Berman et al., 2012; Van den Berg et al., 2016; Kotera et al., 2022). It also improves cognition, including enhanced concentration and restoration of mental function (Kaplan and Kaplan, 1989; Berto, 2005; Berman et al., 2008, 2012).

There also appear to be improvements in psychosocial impacts of NT but this has have received little research attention (Hartig et al., 2014). A meta-analysis of community garden interventions demonstrated moderate improvements in perceived social support, community cohesion, and loneliness (Edwards and Beck, 2002; Spano et al., 2020). Other studies have also demonstrated that green spaces in neighborhoods improve psychosocial wellbeing, for example, perceived neighborhood greenness predicts social cohesion (sense of community, social support) (Sugiyama et al., 2008; De Vries et al., 2013).

Importantly, NT can increase subjective wellbeing over and above the positive effects of physical exercise (Pretty et al., 2007; Nisbet et al., 2009; Gladwell et al., 2013; Araújo et al., 2019; Brymer et al., 2020). This is important as exercise has barriers for individuals with MI that may not be present in NT, including self-consciousness, poor self-efficacy, fatigue, fear of injury, and existing physical injuries/restrictions (Firth et al., 2016). NT interventions can be substantially different to Green Exercise interventions and can overcome many of these issues, for example Forest Therapy can be conducted on wheelchair-accessible paths (Kotte et al., 2019). Some NT interventions do include incidental exercise, for example walking short distances when bird watching but the primary mechanism of therapeutic action is exposure to nature and physical activity is secondary, minimal, and/or optional (Christie and Cole, 2017; Kotte et al., 2019).

Theories explaining the mechanisms by which NT improves wellbeing include Attention Restoration Theory (ART) (Kaplan and Peterson, 1993; Kaplan, 1995), Biophilia (Wilson, 1984), and Stress Reduction Theory (SRT) (Ulrich et al., 1991). ART suggests that some environments are more conducive to restoring mental fatigue resulting from everyday urban lifestyles. The natural world restores cognitive resources and the subsequent ability to focus because attention is held with reduced requirement of effort. A critical review of ART found only partial evidence for the efficacy of ART as an explanatory model as it only demonstrated impacts on executive abilities (Ohly et al., 2016) but not the biopsychosocial benefits of interacting with nature (Hartig and Jahncke, 2017). SRT also considers the impact of metropolitan living and claims that humans have an evolutionary connection with nature and that specific characteristics of nature (complexity, depth, absence of threat) provides restorative benefits (Ulrich et al., 1991). Biophilia notes that humans innately desire connection to life and life-like processes, and exposure to adaptive natural features (e.g., food/water/shelter) is fundamental to biopsychosocial wellbeing (Kellert, 1997). While the SRT and Biophilic frameworks have made a considerable contribution to our understanding of the relationship between human beings and nature, critics suggest they are limited as, for example, they largely overlook the complexity of the relationship between humans and nature, which is inherently multi-dimensional, interactive and multi-sensorial (Joye and Van den Berg, 2011; Brymer et al., 2014; Franco et al., 2017; Araújo et al., 2019).

Despite these potential benefits, peer-reviewed scientific evidence for GSP for MI is sparse. Most extant programs are not formally evaluated or peer-reviewed, with methods and outcomes not published or published in non-scientific documents such as by private consultancy firms or in government reports (i.e., “gray literature”). For example, the UK NHS funds several GSP programs (Rubio et al., 2010; Bragg and Leck, 2017). These include the West Leeds Patient Empowerment Project (The National Health Service [NHS], 2015, 2016) which provided NT interventions such as woodland walks and environmental conservation groups for 5 years (2011–2016) with over 4,000 participants with non-communicable diseases including MI. While the program demonstrated improvements in participant physical and mental health, the evaluation by the private consulting firm Kier Business Services Limited (2016) was not peer-reviewed or published in a scientific journal. This lack of peer review and scientific rigor is unfortunate as this large sample had the potential to provide important evidence for GSP. This lack of good quality peer-reviewed evidence for GSP for adults with MI is unfortunate given gray literature indicating the potential of GSP to be an effective, affordable, and scalable intervention for MI.

Thus, this review is both timely and important; by systematically examining peer-reviewed evidence to identify and critique the characteristics of intervention design and research methods we can improve future GSP research, clinical practice, and participant outcomes in this burgeoning field.

### Aim and research question

This review aimed to identify and critique peer-reviewed studies of adults with MI who live in the community (Population) attending GSP programs (Intervention) in order to create recommendations for future research and clinical practice. Included studies assessed any/all aspects of biopsychosocial wellbeing (Outcomes), were not required to include a control group (Comparator) and could comprise any study type (Study Design).

The research questions for this review are: (1) what types of peer-reviewed evidence exists for GSP for adults with MI living in community settings; (2) what is the quality of this evidence; (3) what are the common findings of this evidence; and (4) what are the implications for future research and clinical practice?

### Objectives

The objective of this review is to systematically identify, describe, review, and compile peer-reviewed evidence to produce recommendations for research and clinical practice. We use a scoping review methodology which is most appropriate for research, as is the case here, that is limited, complex and heterogenous (Peters et al., 2020).

## Methods

### Protocol

The protocol for this review was developed according to the Joanna Briggs Institute methodological guidance for the conduct of scoping reviews (Peters et al., 2020) and the PRISMA-ScR extension for scoping reviews checklist (Tricco et al., 2018). It was produced by author TT and reviewed by all authors; the completed checklist is available as Supplementary material.

### Eligibility criteria

#### Inclusion criteria

For inclusion, publications were required to report primary research published in peer-reviewed scientific journals in English from any country published on/after 1 January 2000. Participants were adults (18+ years) of any gender living in the community with MI; the intervention was any GSP; a comparator was not required nor restricted; outcomes included any biopsychosocial measures, and any/all study designs were included. For the purposes of this review MI was defined broadly and included disturbance in cognition, emotional regulation, behavior, psychosocial abilities, or other mental state associated with significant distress or impairment in functioning (World Health Organisation [WHO], 2022). Inclusion criteria are summarized in Table 1.

**TABLE 1:** Scoping review inclusion and exclusion criteria.

	Inclusion criteria	Exclusion criteria
Publication	Primary research Peer-reviewed journals English language International evidence Published on/after 1 January 2000	Review articles Books, conference proceedings Non-English language Published prior to 2000
Population	Adults Any gender Mental illness	Inpatient
Intervention	Socially prescribed nature therapy	Animal-assisted therapy Exercise as primary therapeutic mechanism
Comparator	No restrictions	
Outcomes	No restrictions	
Study design	No restrictions	

#### Exclusion criteria

Publications were excluded if they did not report peer-reviewed primary research. Review papers were excluded as they are “only as good as the data on which [they are] based” (Charrois, 2015, pp. 145) and as our intention was to identify and evaluate this data ourselves review papers could not provide useful or meaningful sources of information. Non-English publications were excluded due to resource limitations preventing translation. Articles published prior to 2,000 were excluded, thus eliminating outdated/incomparable studies where the classification, diagnosis, and treatment of MI was substantially different. Children (under 18) were excluded as MI in children presents and is treated differently (MFMER, 2002). Inpatient populations were excluded as this review focused on community-based interventions. Animal-assisted therapy was excluded as the main therapeutic action is different to that of NT, being theorized to involve perceived attachment to the animal and oxytocin release in response to caregiving and physical contact (Julius et al., 2012). Furthermore, animal-assisted therapy is resource-intense and not widely available (The Australian National University, 2019), and is thus not consistent with the goals of this review which aims to identify potentially scalable and affordable community-based interventions. Research examining the impact of physical activity as the primary therapeutic mechanism for MI are extensively reported elsewhere and are beyond the scope of this review (Rosenbaum et al., 2014; Stubbs et al., 2018). Publications that were explicitly exercise-based were excluded at title and abstract-review stages, whereas those with ambiguity were reviewed at a full-text level. For studies where the primary therapeutic action was still unclear 3 authors TT, MT, and DD’A initially reviewed the full-text and if questionable, studies were assessed by discussion with the whole research team.

### Information sources

Twelve electronic databases were searched on 4 February 2021 (updated on 15 October 2022) for primary research; these were Academic Search Premier, APA PsycArticles, APA PsycINFO, CINAHL, Cochrane Library, Google Scholar, JSTOR, ProQuest, PubMed, Science Direct, Scopus, and Web of Science. Reference lists of included studies and related review papers were scanned, and an expert in the field of SP and NT (author JB) was consulted to identify additional studies. Databases were selected to provide comprehensive coverage of source material from health and human sciences.

### Search strategy

The search strategy was drafted by author TT in consultation with an expert university librarian, piloted to ensure known studies were included, and reviewed by all authors. Key concepts covered by search terms included SP, MI, NT, GSP, and Blue SP (NT programs that focus on outdoor water environments) (Britton et al., 2018); see Table 2 for the complete search strategy for PubMed.

**TABLE 2:** Search strategy for PubMed database indicating concept search steps, combinations, and wildcards.

Search	Concept	Keywords
1	Social prescribing	“Social prescri*” or “social referr*” or “community referr*” or “community link*” or “link worker*” or “community connect*” or “community navigat*” or “refer to community” or “referral to community” or “non#medical referr*” or “non#medical prescri*” or “health train*” or “well#being program”
2	Mental health	“Mental health” or “mental ill health” or “mental illness” or “mental disorder” or “mental fatigue” or “psychiatric” or “psychiatric illness” or “psychological” or “psychological illness” or “stress” or “depression” or “anxiety” or “recovery” or “low mood” or “well#being” or “quality of life”
3	Nature therapy	“Eco#therap*” or “nature#therap*” or “green#therap*” or “green#care” or “plant#therap*” or “horticulture#therap*” or “therapeutic horticulture” or “community farm*” or “community garden*” or “garden therap*” or “Therapy garden” or “farm therapy” or “care farm” or “conservation therap*” or “forest#therap*” or “forest#bathing” or “shinrin#yoku” or “wilderness therap*” or “adventure therap*” or “urban space therap*” or “Urban green space*” or “green#space” or “agriculture therap*” or “therapeutic agriculture” or “blue therapy” or “blue care” or “urban blue space” or “blue space” or “hydro#therapy” or “aquatic#therapy” or “aqua#therapy”
4	Green/blue prescription	“Park#prescri*” or “green#prescri*” or “blue#prescri*”
5	All searches combined	(Social prescribing and mental health and nature therapy) OR [(green/blue prescription) and mental health]
6	Limits	English language 2,000 onward Peer-reviewed journal articles

^#^Indicates zero or one characters, *indicates one or more characters.

Search terms were developed based on known SP and NT studies and review articles. Search terms regarding blue prescriptions were adapted from a recent blue care review paper (Britton et al., 2020), however, search terms from this review relating to blue exercise activities (e.g., surfing) were excluded. The use of Mesh headings, search terms, wildcards, and limits were adapted to each database. Wildcards ensured all permutations of words were included, and broad search terms captured country-specific vernacular. Concept searches were conducted individually and then combined.

### Selection of sources of evidence

All database searches were conducted by author TT with results imported into Endnote X9 (Clarivate, 2013) and duplicates removed. Initial refinement of potential papers according to selection criteria was completed based on title screening, followed by abstract screening of remaining papers (author TT). Full-text review of remaining papers was conducted independently by 3 authors (TT, MT, and DD’A) and disagreement resolved by discussion.

### Data charting and items

A data charting tool (data extraction tool specific to scoping reviews) was developed in Microsoft Word (Microsoft, 2022) based on the template by Peters et al. (2017). This tool included the Study Setting, Design, Participant Characteristics, Intervention (and Control), Outcome Measures, and Findings. Piloting of data extraction on 2 papers elucidated the necessity of including an additional data point (i.e., Data Collection). Charting was conducted independently by 2 authors (TT and MT) and inconsistencies resolved with discussion. Study authors were emailed to gain additional required information.

### Critical appraisal of evidence

Appraisal was conducted independently by 2 authors (TT and DD’A) using the Mixed Methods Appraisal Tool (MMAT; Hong et al., 2018). A consensus approach was also used to confirm the ratings according to the 5 criteria of the MMAT. A scoring system of 1 (no criteria met) to 5 (all criteria met) was used independently by 2 authors (TT and DD’A) and inconsistencies resolved with discussion.

### Synthesis of results

Charted study results were imported into a separate Microsoft Word table (Microsoft, 2022) organized by study. Common findings across studies were identified and color-coded by author (TT) and re-tabulated by result. This synthesized results table was checked against full-text papers to ensure accuracy. Frequencies were calculated.

## Results

### Study selection

**FIGURE 1:**
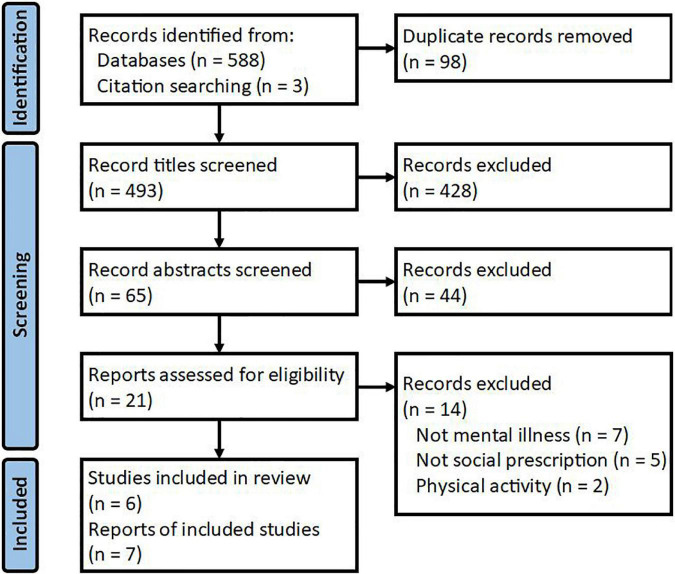
Scoping review study selection PRISMA diagram. Adapted from Page et al. (2021).

Database searches yielded 588 publications and reference list checking another 3; 98 duplicates were removed leaving 493 unique publications (Figure 1). Next, title screening excluded 428 publications, primarily because studies were exercise-based or not SP. Sixty-five abstracts were screened and 44 excluded for the same reasons. Twenty-one-text publications were reviewed and 14 excluded as they were not MI (*n* = 7), not SP (*n* = 5), or were exercise-based (*n* = 2). Harris et al. (2014) were contacted to confirm participants’ mental health status and referral process. Seven publications were included in this review reporting 6 unique studies; these were Wilson et al. (2010, 2011), Harris et al. (2014), Christie and Cole (2017), Maund et al. (2019), McEwan et al. (2019), and Thomson et al. (2020). One study was described in 2 publications separated into qualitative (Wilson et al., 2010) and quantitative (Wilson et al., 2011) components.

Study selection elucidated the relative lack of peer-reviewed research in this area. Given the number of large GSP programs currently being conducted internationally, the lack of rigorous research was stark. This represents a lost opportunity to demonstrate the efficacy of such programs and thus capacity to gain funding and expand such programs. Additionally, current/future programs are not being adapted to improve their efficacy as additional evidence could allow. Contrastingly, evidence of limited efficacy could prevent diversion of funding to other programs; however, under-publication of negative results “Publication Bias” (Ayorinde et al., 2020) could prevent this regardless of sufficient peer-reviewed evidence.

### Quality of included studies

Overall, the quality of reported studies was excellent, as assessed on the MMAT; scores are presented in Table 3. All studies had a clear research question and corresponding findings (MMAT screening item) and are thus excluded from Table 3 for brevity.

**TABLE 3:** Study methods and results summary.

References	Setting	Design	Participants	Referring service(s)	Intervention	Data collection and analysis	Findings
Christie and Cole, 2017	Northwest England, United Kingdom Woodland homestead	Ethnographic	Mental illness • *n* = 7 volunteers (86% female) • *n* = 2 property owners (50% female) • All attending for 1+ years • 35–67 years old	• Local charity organization • Others (unspecified)	Therapeutic horticulture Format • 6 wks • 2 sessions of 4–6 h/wk • Groups of 9 Activities 1. Gardening 2. Construction e.g., bird feeders 3. Maintenance e.g., painting	Procedure • Semi-structured interviews Questions 1. Motivation to attend. 2. Feelings about the intervention environment. 3. Personal meaning of participation. Analysis • Thematic	Themes 1. Beneficial intervention environment e.g., tranquil, sense of escape, *“in and with nature”* 2. Social connectedness, meaning and belonging 3. Meaningful tasks, learning new skills, sense of purpose
Harris et al., 2014	Logan, QLD, Australia community food garden	Qualitative	Mental illness • *n* = 12 African humanitarian migrants • All attending for 1+ years	Multicultural-specific community referral service	Allotment gardening Format • 20 m^2^ plot/participant • Participants attend 4–5 times/week	Procedure • Semi-structured interviews Questions 1. Motivation to join. 2. Motivation to continue attending. 3. Why is involvement in the garden personally important? Analysis • Thematic	Themes 1. Land tenure, symbolism of connection to land 2. Farming is central to migrant culture 3. Community belonging
Maund et al., 2019	Gloucestershire, England, United Kingdom Inland Wetland	Mixed methods Quasi experiment one-group pretest-post-test Qualitative	Anxiety, depression • *n* = 16 patients (50% female) • *n* = 2 mental health support workers • ∼51 years old	NGO mental health workers	Wetland exploration and education Format • 6 wks • 2 h/wk • Groups of 8–10 Activities • Guided nature walks (0.8–1.5 km) • River walk (2.5 km) • Fauna watching and education (birds, otters) • Canoeing	Quantitative Procedure • Self-report questionnaires Questionnaires • Wellbeing (WEMWBS) • Stress (PSS) • Anxiety (GAD-7) • Affect (PANAS) Analysis • Wilcoxon signed rank test – Qualitative Procedure • Focus groups • Semi-structured interviews Questions 1. Changes in mental health. 2. Contribution of wetland environment to mental health. 3. Contribution program design to mental health. Analysis • Content analysis	Quantitative • Sig. improvements on all measures – Qualitative • Improved affect (i.e., peaceful environment, distraction) • Decreased social isolation • Improved confidence • Feel healthier
McEwan et al., 2019	Sheffield, England, United Kingdom Metropolitan City	RCT	Mental illness: recovering quality of life scale score in clinical range • *n* = 164 adults (60% female)	Social prescription by GPs (*n* = 59) • Social media • Pamphlets and flyers • Wildlife trusts • Council staff • Large local employers	Smartphone app prompts participants to notice good things Format • 1 wk • 1 SMS Prompt per day • Individual Intervention • “Green” Condition • Smartphone app uses GPS and prompts to “enter 1 good thing you notice” when near urban green space. Control • “Built” condition • As per Green but prompts are random.	Procedure • Self-report questionnaires Questionnaires • QoL (ReQoL) • Positive Affect (TPAS) • Nature Connectedness (INS and NR) • Time spent outside as a child • Time spent outside in last year Analysis • MANOVA	Both conditions • Sig. improvement all scores • No main effect of condition • Greater improvement in QoL in participants with mental illness Built condition • Greater improvement in QoL if less time outside in last year Green Condition • Greater increase in Nature Connectedness if greater time spent outside as a child, or less time spent outside in the last year • Lower baseline Nature Connectedness predicts greater improvement • Improvements in Nature Connectedness and Relaxed Positive Affect predict improvement in wellbeing
Thomson et al., 2020	Manchester, England, United Kingdom Art Gallery and Park	Mixed methods Qualitative Quasi experiment one-group pretest-post-test	Mental illness or disadvantaged • *n* = 46 (50% female) • 53 years old (range 44–70)	Community mental health nurses • Day center for disadvantaged and vulnerable adults	Arts and nature-based activities in a park and gallery Format • 10 wks • 2 h/wk • Groups of 16–26 Activities • Gardening • Museum art and nature-focused activities e.g., painting nature scenes, printing and drying flowers	Qualitative Procedure • Observation • Semi-structured interviews • Participant diary entries Analysis • Inductive thematic – Quantitative Procedure • Self-report questionnaires Questionnaires • Mood (UCL-MWM) • Affect (PANAS) Analysis • Paired *t*-test	Qualitative Themes 1. Improved self-esteem, skill acquisition, confidence 2. Decreased social isolation, improved interpersonal confidence 3. Formation of community, shared experience of mental illness – Quantitative Wellbeing • Sig. improved
Wilson et al., 2010	Glasgow and Clyde, Scotland, United Kingdom Woodland	Qualitative	Mental illness • *n* = 28 clients (36% female) • *n* = 8 clinicians	Community mental health services • Mental health employment services • Forensic services • Other tertiary services	Ecotherapy format • 12 wks • 3 h/wk • Groups of 6–12 Activities • Conservation e.g., weeding • Construction e.g., bird box • Bushcraft e.g., map reading • Exercise e.g., Tai Chi, • Environmental art e.g., photography • Other e.g., Scottish Museum of Rural Life	Procedure • Interviews • Focus groups Questions 1. Program outcomes 2. Facilitators of change (service characteristics) Analysis • Interpretative phenomenological analysis	Client outcomes 1. Improved fitness, physical, and mental health 2. Daily structure important 3. Transferable knowledge and skill 4. Increased socialization 5. Participation with clinicians improves therapeutic relationship 6. Meaningful work leads to pride and self esteem 7. Ongoing community engagement e.g., volunteering, higher education
Wilson et al., 2011	As above	Quasi experiment one-group pretest-post-test	Mental illness • *n* = 77 clients (26% female)	As above	As above	Procedure • Self-report questionnaires Questionnaires • General health: (SF12v2) • Wellbeing (WEMWBS) • Physical activity (SPAQ) analysis • Paired *t*-tests	General health • No sig. changes Mental wellbeing • No sig. changes Physical activity • Sig. increase

QoL, quality of life; Sig., significant.

Questionnaires. GAD-7, Generalized Anxiety Disorder Questionnaire (Spitzer et al., 2006); INS, Inclusion of Nature With Self Scale (Schultz et al., 2004); NR, Nature Relatedness Scale (Nisbet et al., 2008); PANAS, Positive and Negative Affect Schedule (Watson et al., 1988); PSS, Perceived Stress Scale (Cohen et al., 1983); ReQoL, Recovering Quality of Life Scale (Keetharuth et al., 2018); SF12v2, Short Form 12, Version 2, Health Survey (Ware et al., 2007); SPAQ, Scottish Physical Activity Questionnaire (Lowther et al., 1999); TPAS, Types of Positive Affect Scale (Gilbert et al., 2008); UCL-MWM, UCL Museum Wellbeing Measure (Thomson and Chatterjee, 2015); WEMWBS, Warwick–Edinburgh Mental Well-Being Scale (Parkinson, 2006).

The overall good quality of included studies was surprising as reporting of methods, in particular intervention information and participant characteristics, was poor (discussed further below). However, the focus of the MMAT is the appropriateness and congruence of the research design and analysis in terms of the research question (i.e., methodological rigor). For example, participant characteristics in Harris et al. (2014) were poorly reported but the research question was clear, data collection and analysis were appropriate to answer the question, and the findings/themes were well supported by quotes from participants.

### Summary of included studies

The following section outlines (1) learnings from the findings of GSP studies included in the paper, and (2) learnings from research that examines the process and outcomes of GSP. A summary of the methods and findings of all included studies can be seen in Table 4.

**TABLE 4:** Mixed methods appraisal tool quality appraisal scores by study design components.

References	Qualitative	RCT	Non-randomized	Mixed methods
	**Maximum possible score**			
	5	5	5	5
	**Quality scores**			
Christie and Cole, 2017	5			
Harris et al., 2014	5			
Maund et al., 2019	5		5	5
McEwan et al., 2019	5	4		
Thomson et al., 2020	5		5	5
Wilson et al., 2010	5			
Wilson et al., 2011	5			

### Findings of included studies

All 6 included studies reported improvements in participant biopsychosocial wellbeing, however, these improvements varied in terms of quality and quantity in each domain. A detailed breakdown of these findings can be seen in Table 5; for brevity, only study-specific results that are not presented in Table 4 are individually referenced in the result synthesis sub-sections below.

**TABLE 5:** Study results for biopsychosocial domains and environment factors.

References	Global wellbeing	Physical	Psychological								Social						Nature		
					
			Psychological wellbeing	Positive affect	Self-esteem and self confidence	Self -identity	Skill acquisition and development	Building and creating	Achievement and pride	Routine and structure	Social isolation	Social connection	Mutually supportive relationships	Teamwork	Shared experience of mental illness	Social skills and confidence e.g., communication	Connection to nature	Fascination with fauna and flora	Beauty of environment
Christie and Cole, 2017		↑	↑	↑	↑	↑	X	X	X	X	X	↑	↑	X	X		X	X	X
Harris et al., 2014	↑		↑	↑	↑	↑	X	X	X		X	↑	↑	X	X Other migrants		X		
Maund et al., 2019		↑	↑sig	↑sig	↑		X		X		X	↑			X	↑	X	X	
McEwan et al., 2019	↑sig			↑sig													↑sig		No change
Thomson et al., 2020	↑sig		↑	↑	↑	↑	X	X	X	X	X	↑	↑	X	X	↑	X		
Wilson et al., 2010		↑	↑	↑	↑		X	X	X	X	X	↑	↑	X		↑			
Wilson et al., 2011		General health non-sig Physical activity ↑sig	Non-sig																

All results presented are qualitative unless (non)significance is indicated. X indicates a factor participants experienced, ↑ indicates a factor participants reported increased.

#### Global quality of life and wellbeing

All 6 studies reported improvements in at least 1 of physical, psychological, and/or social QoL or a wellbeing domain (elaborated below) but only 3 studies assessed global/overall QoL and wellbeing, defined herein as “an individual’s perception of their position in life in the context of the culture and value systems in which they live and in relation to their goals, expectations, standards and concerns” (WHOQOL Group, 1993). These included 2 quantitative studies that assessed quality of life/wellbeing using a total score on a QoL/wellbeing measure. McEwan et al. (2019) reported significant improvements in overall scores for the Recovering Quality of Life for people with MI questionnaire (ReQoL) (Keetharuth et al., 2018), and Thomson et al. (2020) reported significant improvements in overall wellbeing on the UCL Museum Wellbeing Measure (Thomson and Chatterjee, 2013). Additionally, 1 participant in qualitative study (Harris et al., 2014) noted *“it is very important for me to be in the garden for my whole total wellbeing”* (p. 9209). McEwan et al. (2019) found greater improvements in QoL were present for those living with MI (versus not living with MI) and for those having spent lower levels of time outside in the year preceding the intervention.

#### Physical wellbeing

Although this review specifically excludes exercise interventions as they have barriers to participation (Firth et al., 2016), physical wellbeing can and does improve from exposure to nature (Twohig-Bennett and Jones, 2018) and is thus included in this review paper. Three studies reported qualitative improvements in participant perceived physical wellbeing, including sleep quality, vigor, fitness, pain, symptom management, weight loss, and breathing under exertion. Despite participant reports, Wilson et al. (2011) did not find significant quantitative improvements in self-reported physical health as measured by the SF-12v2 Health Survey (Kosinski et al., 2007). Participants did, however, report significantly increased weekly moderate exercise from pre to post-intervention. During qualitative interviews 1 participant stated they now left the house more often and completed more activities suggesting increased incidental exercise (Wilson et al., 2010, 2011). A participant in Maund et al. (2019) stated the incidental exercise during the intervention was enjoyable as it was not formal or mandated.

#### Psychological wellbeing

Five studies reported improved overall psychological wellbeing, including symptoms of anxiety, depression, and panic. While the quantitative study by Maund et al. (2019) demonstrated a significant improvement in wellbeing, changes reported by Wilson et al. (2011) did not reach significance. All 6 included studies provided evidence of increased positive affect (e.g., happiness), including 2 studies that found significant improvements using quantitative analyses (Maund et al., 2019; McEwan et al., 2019). Maund et al. (2019) also found a significant decrease in negative affect (e.g., anger). In 2 studies (Christie and Cole, 2017; Maund et al., 2019) participants described the interventions as relaxing, a distraction from negativity, and providing a sense of escape, with 1 participant stating it was a *“break from reality, a 2-h holiday”* (Maund et al., 2019). In these studies participants reported that prior to the intervention they spent most of their time home alone, sometimes in unpleasant conditions (e.g., without electricity), which 1 participant described as a *“bleak existence”* (Christie and Cole, 2017). In 3 studies participants reported the interventions provided something to look forward to and a reason to get out of bed or leave the house, and that the routine and structure of the program contributed to their psychological wellbeing.

Five studies demonstrated improvements in participant self-esteem and self-confidence, with these largely related to activity-based skill development. Two studies reported improved agency (Christie and Cole, 2017; Thomson et al., 2020), 1 improved self-reliance (Harris et al., 2014), and 3 a better sense of self-identity, with 1 participant describing changes as resulting from overall improvements in perceived self-worth (Thomson et al., 2020). Participants reported satisfaction when they learned, developed, and taught others new skills (e.g., growing crops, constructing bird boxes), with participants using newly developed skills to be productive and complete what they considered meaningful tasks, resulting in a sense of purpose, achievement, and pride (Wilson et al., 2010; Harris et al., 2014; Christie and Cole, 2017; Thomson et al., 2020).

#### Social wellbeing and capital

The majority of studies (*n* = 5) reported that prior to the intervention participants were experiencing extreme social isolation. Participation facilitated social connection (*n* = 5), development of mutually supportive relationships (*n* = 4), and feelings of community, meaning, and belonging (Harris et al., 2014; Christie and Cole, 2017). Participants in most studies (*n* = 4) were required to cooperate on tasks and reported these shared experiences and goals *“facilitated quick bonding”* (Wilson et al., 2010); participants *“looked forward to seeing each other”* (Christie and Cole, 2017) and they assisted in creating *“meaningful and lasting relationships”* (Maund et al., 2019).

Participants in 4 studies reported it was beneficial to engage with others with a shared experience of MI (Harris et al., 2014; Christie and Cole, 2017; Maund et al., 2019; Thomson et al., 2020). For example, 1 participant stated *“I think a lot of us probably feel like we don’t really fit in, I think here we just understand each other”* (Maund et al., 2019) and another said *“it was very important to relate to people, that we had a common ground factor, and that was our mental health experiences*… *I would never be able to have the same chats and the same connection and the same understanding and empathy”* (Thomson et al., 2020).

Participants in 3 studies also reported the intervention helped them to develop social skills and confidence (e.g., communication skills). One study (Wilson et al., 2010) conducted follow-up assessment and reported some participants had commenced other support programs, volunteering work, and higher education. Similarly, participants in the study by Harris et al. (2014) reported that experiences in the community garden had helped develop friendships outside the garden due to shared interests in farming.

#### Connection to nature

Participants in 5 studies reported experiencing a profound connection to nature and the earth, saying things like *“it’s something bigger than myself to be connected to”* (Maund et al., 2019). In 2 studies participants’ developed new interest and confidence in accessing nature (Wilson et al., 2010; Maund et al., 2019), with 1 participant explaining that the intervention had *“opened their eyes to nature and what was available on their doorstep”* (Wilson et al., 2010). McEwan et al. (2019) considered 2 separate but related models in explaining these connections; the Nature Relatedness model (NR) (Nisbet et al., 2009) which describes “one’s appreciation for and understanding of our interconnectedness with all other living things on the earth” (p.718). Nature Connectedness (NC) which refers to an emotional, cognitive, and experiential connection with the natural environment (Mayer and Frantz, 2004). Increased noticing of nature in McEwan et al. (2019) significantly increased both NR and NC.

In 2 studies showing improved psychological wellbeing, participants attributed decreased anxiety to the natural environment which they described as peaceful, relaxing, tranquil, and calm (Christie and Cole, 2017; Maund et al., 2019). They also reported the natural beauty and fascination with fauna and flora offered a distraction from their problems. For example, participants in Maund et al. (2019) said *“when you come here you are so engrossed in the animals and birds, all your troubles, they just disappear”* (p. 9) and *“I think walking in the wildflower garden was fabulous for me. All the beautiful variety of plants and flowers, lots of different colors”* (p. 9). Clinicians in this study also reported that participants being distracted by natural stimuli that engaged all 5 senses was beneficial (Maund et al., 2019).

These findings in combination may have implications for the long-term impact of NT interventions for MI. By improving participant confidence in accessing natural environments and assisting them to find new connections to nature and earth, and supporting the appreciation of nature, interventions may become self-sustaining with participants continuing to access natural environments after the intervention and therefore having ongoing benefits. This finding was hinted at in 1 study where a participant stated *“I wouldn’t have come somewhere like this by myself*… *but now I have been because you all [study staff] supported me I think I would feel better about coming here again. Maybe not by myself but with a friend”* (Maund et al., 2019). Even in the absence of continued NT, elements of Behavioural Activation (BA) and social connection associated with NT interventions may be sustained after the intervention. In support of this, Wilson et al. (2010) provided evidence of increase BA with participants leaving the house more often, completing more activities, and some commencing volunteering and education at follow-up. Improved social relationships was a consistent theme across studies, with 1 participant noting a clear link between natural environment and social isolation (Christie and Cole, 2017) which is consistent with our evolved innate desire for group inclusion and social interaction (Buss, 2019).

Social BA may be linked to participants experiencing social interaction in natural environments, creating feelings of familiarity and safety due to the calming effect of nature, as proposed by ART (Kaplan, 1995). It may also reduce social isolation through non-confrontational nature-based social activities, or simply because humans evolved to interact and gather resources in groups and as such spending time with other people in nature might be an activity that is hardwired (Buss, 2019).

## Research methods and design of included studies

### Setting

Of 6 unique studies, 5 were conducted in the UK and 1 in Queensland, Australia. Settings varied widely including a community garden (Australia), 2 woodland settings (homestead and outdoor), an inland wetland, museum, and metropolitan city. The studies included in this review lacked detailed information about the environmental features across the studies such as types of fauna and flora, still or running water, air quality, and weather. However, the setting of Harris et al. (2014) did facilitate growing culturally important vegetables (cassava, maize) which contributed to the apparent psychosocial impacts of the intervention, giving participants links to their country of origin, and allowing them to share their produce with others.

### Design

Study designs included 2 qualitative (Harris et al., 2014; Christie and Cole, 2017): 3 mixed methods (Maund et al., 2019; Thomson et al., 2020) plus (Wilson et al., 2010, Wilson et al., 2011); and one RCT (McEwan et al., 2019). In general data were collected either during the intervention or immediately post-intervention, with only (McEwan et al., 2019) collecting any follow-up data. For the qualitative studies (Harris et al., 2014; Christie and Cole, 2017) data collection occurred during the intervention. Wilson et al. (2010, 2011), Maund et al. (2019) and Thomson et al. (2020) all conducted quasi-experiments with quantitative follow-up data collected immediately post-intervention, and qualitative straight after this; Thomson et al. (2020) attempted follow-up but only interviewed 1 participant at 3 and 6 months, and did not report this data. McEwan et al. (2019) conducted an RCT with quantitative data collection immediately pre-intervention, 7 days and 1 month follow-up.

### Participants

Across the studies, data on participant characteristics was limited which has direct implications for research. A total of 334 participants were included across the 6 studies (accounting for overlap and dropouts), with samples ranging from 9 to 77 in face-to-face, and 164 in the Smartphone App (McEwan et al., 2019) intervention. Gender balance across studies varied considerably from 26% female (Wilson et al., 2011) to 86% female (Christie and Cole, 2017), although on average about 50% of participants were female across studies. Six papers did not provide full/any age data (participant mean age in Maund et al. (2019) was estimated based on age bracket data provided), and Christie and Cole (2017) had a large age range (35–67 years). Treatment response such as ethnicity or socio-economic status were poorly/not reported.

### Recruitment

Reporting of recruitment strategies, referring services, and referral type was poor across studies. General information such as “Local Charities” or “Mental Health Workers” was often provided, except for Wilson et al. (2010) where referring services were specified but not described. Across studies, missing information included the size and scope of services and occupation of referrers (e.g., social workers versus faith-based community leaders), which has implications for participant characteristics such as severity of MI, multi-morbidity, and/or dual diagnosis.

### Interventions

There was large variability in the format of interventions, including the time over which the intervention was conducted. The evaluation of GSP interventions that are conducted for as little as 1 week (McEwan et al., 2019) to over 1 year (Christie and Cole, 2017) has implications for practice and research. Face-to-face interventions usually ran from 6 to 12 weeks (*n* = 3) and were conducted in groups of approximately nine participants (*n* = 3), but the largest had over 45 participants (Harris et al., 2014). There was some overlap in activities across interventions; 4 interventions included horticulture such as gardening and weeding, 2 interventions included construction activities including small objects (e.g., bird boxes) and larger items (e.g., dry stone walls around garden beds), 3 included art and decorating activities, and 2 interventions included museum visits. Activities unique to specific interventions included canoeing (Maund et al., 2019) and the broad range of activities in Wilson et al. (2010) such as bush craft (e.g., orienteering, shelter building, campfire cooking).

Although there was variability in intervention types many shared hypothesized therapeutic mechanisms similar to those framed by nature connection theories. For example, therapeutic mechanisms in horticulture include completing meaningful and enjoyable tasks, community connection, social connection though shared experiences, and local environment connection (Relf, 1999; Shinew et al., 2004; Husk et al., 2016). These mechanisms of action were evident across all included studies (i.e., 5 included meaningful tasks, 5 included social connection, and 5 increased connection to nature).

In addition to variability in format and tasks, a potential confounder across studies was physical activity which evidence suggests can mediate the impact of NT on MI (Bélanger et al., 2019). However, in all studies the primary activity was exposure to nature and exercise was incidental. For example, in Maund et al. (2019) participants completed activities such as bird watching over an 800 m walk in 2 h; this speed is 8% of average adult walking pace and well below the required “brisk” pace (for any age group) where physical and mental health status are likely to improve (Ainsworth et al., 2011). Similarly, in Christie and Cole (2017), despite being referred to as an “exercise intervention” by the authors, participants could choose to complete non-exercise activities, and all of their included activities (e.g., gardening) were classified as nature-based rather than exercise-based in all other included studies. Additionally, in McEwan et al. (2019) the smartphone intervention did not affect participant physical activity and significantly improved wellbeing. Furthermore, participants frequently reported improved wellbeing from relaxing during non-exercise activities (i.e., viewing fauna and flora improved their mood).

### Evaluations

These findings are associated with research and describes the quality of evaluations being undertaken in the included studies.

#### Qualitative evaluations

Trustworthiness of qualitative research indicates the meaningfulness and usefulness of results, also conceived of as the validity and relevance of results (Mays and Pope, 2000). The quality of evaluations was assessed according to the procedure of Nowell et al. (2017) as it is widely used and accepted as an appropriate assessment of quality (Stenfors et al., 2020). Quality was assessed based on the credibility, dependability, transferability, and confirmability of the research (Nowell et al., 2017). Trustworthiness of analysis was only explicitly considered by Christie and Cole (2017) who addressed the credibility of their findings by specifying that all authors independently generated codes/themes and analyzed data. Harris et al. (2014) did not explicitly mention trustworthiness but credibility was partially addressed as initial codes/themes were generated by one researcher which data was re-analyzed by a second. No other studies reported procedures that compared “triangulated” theme generation or analysis (Nowell et al., 2017).

Dependability i.e., replicability (Nowell et al., 2017), was limited across studies which all used inductive analyses but poorly reported any further details. Good quality qualitative research should provide detailed information about the research team, study design, data analysis, and findings (Tong et al., 2007) and this reporting was varied. For example, while the theoretical framework was well reported, interview settings and data collection methods (including questions and prompts) varied across studies, thereby limiting replicability and critique of study methods (Tong et al., 2007).

Transferability of findings to other populations/settings (Nowell et al., 2017) was limited by poor reporting of participant characteristics, sample sizes and consideration of data saturation (no new emerging themes) (Marshall et al., 2013).

Confirmability (neutrality of findings) across studies was difficult to assess as interviewer characteristics (gender, occupation, training, etc.) were not reported; this limits assessment of bias from researcher’s perspective such a background and so on (Tong et al., 2007; Nowell et al., 2017). Nonetheless, studies provided clear findings and sufficient participant quotes to provide context, which are central in determining study quality (Hong et al., 2018). This suggests that that despite limited reporting it is reasonable to assume the findings are at least somewhat trustworthy, and that future research should investigate these themes further.

#### Quantitative evaluations

Two studies (Maund et al., 2019; Thomson et al., 2020) used the Positive and Negative Affect Scale (PANAS; Watson et al., 1988) and 2 used the Warwick Edinburgh Mental Wellbeing Scale (WEMWBS; Stewart-Brown and Janmohamed, 2008); no other questionnaires overlapped across studies (McEwan et al. (2019) used the ReQoL and Thomson et al. (2020) the UCL Museum Wellbeing Measure). The lack of a standard set of instruments makes it difficult to compare findings across studies and assess the statistical or clinical significance of findings. Small sample sizes and lack of statistical power was a limitation for quantitative studies (Maund et al., 2019; Thomson et al., 2020). Where the sample size of future studies allows, validated and widely used quantitative measures should be utilized.

## Discussion

This review aimed to identify and critique peer-reviewed evidence for GSP for MI and produce recommendations for research and clinical practice. Results indicated a lack of peer-reviewed evidence in the area and generally poor reporting of research methods and outcomes in existing literature. However, included studies suggested that GSP may improve biopsychosocial wellbeing and connection to nature.

Study designs included 1 RCT with random assignment and independent variable manipulation with all others quasi-experiences and/or qualitative studies. Quantitative components of these studies were all immediately post-intervention and provided evidence for significant improvements in wellbeing. Whilst traditional thinking would suggest that the limited number of “Gold Standard” RCTs may make reliable evaluation of study outcomes difficult, it is not necessarily a problem. Evidence suggests that RCTs can be subject to design flaws, for example incorrect follow-up periods (Paraskevas et al., 2019) which is relevant here given the lack of follow-up in all but 1 study (McEwan et al., 2019) and lack of an established standard follow-up period in this area of research (Oh et al., 2017; Spano et al., 2020). Similarly, RCTs are not always representative of reality (Paraskevas et al., 2019) which may be true here as limited extant evidence in the area could impact content and construct validity, in which case the contribution of the qualitative studies may be more valuable in guiding future programs and research (Jiménez-Buedo and Russo, 2021). Additionally, given the nature of the interventions utilized, traditional RCT research design might prove difficult. For example, Christie and Cole (2017) examined participants that had attended a country homestead for over a year. A 1-year control group is not feasible due to ethical issues in delayed intervention for a waitlist control, limited resources, and lack of established “treatment as usual” for an active control in this emerging area of research. Additionally, the frequent use of retrospective evaluation design negates the opportunity for RCTs and limits opportunity for quantitative analysis. However, the qualitative studies provided rich data into the mechanisms of action and potential areas of improvement.

Qualitative evaluation methods were assessed using the widely accepted credibility, dependability, transferability, and confirmability framework (Sparkes and Smith, 2009; Nowell et al., 2017); in general, these aspects of the qualitative studies were poorly reported. Quantitative evaluations were limited by small sample sizes and a lack of standard set of instruments. However, assessment using the MMAT indicated that the studies were of overall good quality. For each study the research question was clear, data collection and analysis were appropriate to answer the question, and the findings/themes were well supported by quotes from participants. This suggests that despite limited reporting it is reasonable to assume the findings are at least somewhat trustworthy, and that future research should investigate these themes further.

Recruitment and referral strategies were also poorly reported, which has implications for practice and research. SP varies from signposting to link workers providing wraparound care (Kimberlee, 2015) but without this information reported it is impossible to know which referral pathways are effective for which groups of participants, and thus specific recommendations for future research and clinical practice are limited.

All studies reported improvements in psychological wellbeing including, mood, self-confidence, and self-worth, with participants indicating that a sense of escape and skill development were important in these improvements. Participants in most studies also reported reduced loneliness and social isolation, improved sense of community, meaning, and belonging. A recent study by Dobson et al. (2020) of an allotment gardening intervention in the UK duplicated these findings whereby participants shared food, knowledge and skills, and reported high social cohesion and a sense of community. This finding is also supported by a recent meta-analysis where horticulture interventions significantly improved psychosocial wellbeing (Spano et al., 2020), and more broadly, a review that found that even in urban settings NT interventions can improve social connectedness (Leavell et al., 2019).

Participants also reported improved social skills and confidence which appears to be in part due to a shared experience of MI that made participants feel more comfortable and confident interacting in groups, as though they were part of a safe “in-group,” for example saying they “*just understand each other*” (Maund et al., 2019) and “*had a common ground factor*” (Thomson et al., 2020). These findings are important for MI recovery as opportunities to socialize comfortably as part of the in-group and develop social skills may influence short and long-term outcomes (Hendryx et al., 2009). However, MI diagnoses were very poorly reported across studies allowing limited capacity to assess the relative impact of MI diagnosis in group cohesion. Similarly, poor reporting of demographics was a problem as it limited the ability to assess what in-groups may have formed within interventions, and identify how these could have affected outcomes, for example social isolation tends to increase with age (Fakoya et al., 2020), so the benefits of social interaction may have been more pronounced in older adults.

Despite apparent improvements in biopsychosocial wellbeing across studies, the generalizability of findings is limited due to extensive under-reporting. In particular limited reporting of MI diagnosis was common and is problematic as this can vastly affect the efficacy of interventions (Barlow, 2021) and it is impossible to ascertain the range and severity of MI within and between study samples. For example, McEwan et al. (2019) found greater improvements in QoL for participants with MI versus those without but could not offer any further insights into what works for whom. However, the nature of the populations and referring services of included studies somewhat explains this; for example, community-based welfare workers may identify participants who require intervention but do not have the skills to conduct a formal assessment and diagnosis. Or in the case of the Harris et al. (2014), migrant status was used to infer experiences of trauma and post-traumatic stress disorder but formal assessment of MI was not conducted due to potential to be distressing or (re)traumatizing (Blackmore et al., 2020). Finally, other participant characteristics that may impact treatment response such as ethnicity or socio-economic status were poorly/not reported. This is important as marginalized or disadvantaged groups have lower access to MI treatment, and these groups may have benefited more than others due to variable and somewhat limited existing treatments (Villatoro et al., 2018).

This review found that GSP increased perceived connection to nature which extant evidence indicates correlates strongly with mental wellbeing (Capaldi et al., 2014). Participants across studies delighted in seeing and interacting with fauna and flora which is consistent with the Affordances Framework of Brymer et al. (2020) where the experience of nature is related to existing and potential interactions rather than passive observation (Brymer et al., 2014, 2020). However, participants also reported that mindful emersion was also healing and restorative (Christie and Cole, 2017; Maund et al., 2019) which is consistent with ART (Kaplan, 1995) in which relaxed attention to natural objects (“soft fascination”) results in cognitive restoration as it allows unconscious processing that facilitates psychological healing and reduces cognitive overload (Daniel, 2014). This finding is unsurprising given the enormous body of evidence for the benefits of mindfulness, and its central role in other types of NT, for example Forest Therapy (Kotte et al., 2019). However, these theories are not mutually exclusive as mindful awareness can include all 5 senses i.e., touching, and therefore interacting with, natural objects. Future research could examine the benefits of both passive and active immersion in nature, and the efficacy for different groups of people, e.g., physical capacity for interaction. Similarly, clinical practice would benefit from considering the capacity and needs of clients when choosing more passive or active interventions for referral.

Study settings varying widely and were poorly reported, nonetheless results indicated that GSPs can be successful in a variety of environments. Variability between biomes does impact the feasibility of outdoor interventions, however, and future research could benefit from detailed reporting of intervention environment including inclement weather conditions (Goldman et al., 2020). ART and SRT suggest that beneficial environments should be restorative or unthreatening (Ulrich et al., 1991; Kaplan, 1995), however, other evidence indicates NT can have benefits regardless of biome (Burls, 2007). Therefore, future research needs to examine how the environment mediates the benefits of GSP interventions (for example is a thunderstorm intimidating or exhilarating). The wide variety of settings is consistent with the findings of Hartig et al. (2014) who state that there is no consistent definition of “nature” or “natural environments” in the research and thus NT interventions include anything from remote jungle to pot plants in urban apartments. There are also issues regarding classification of locations, for example even “allotment gardens and urban parks comprise natural features, appear natural, and provide opportunities to engage with and follow natural processes, but they are typically designed, constructed, regulated, and maintained” (Hartig et al., 2014, p.208).

Likewise, variability in type of NT intervention and therapeutic mechanisms (e.g., gardening vs. bird watching) may impact generalizability (Sempik et al., 2003), but given the reported benefits of NT across interventions it is likely exposure to, and engagement with, nature in general rather than specific activities improves wellbeing. The variability and flexibility of GSP activities, including adaptability to local population and biome is a major strength of this area as it can provide appropriate and effective interventions for a wider range of people. For example, the intervention setting of Harris et al. (2014) allowed for growing culturally important vegetables and illustrates the value of GSP in facilitating access and optimizing interventions for local community members, in this case culturally and linguistically diverse minorities (Harris et al., 2014). Similarly, even within local areas biomes can vary considerably as, for example, even small changes in elevation can impact the fauna and flora. This provides further opportunities for GSP to be matched to participant needs, e.g., not referring a client with hydrophobia to a coastal intervention when an inland one is also available.

Results demonstrated improvements in quantitative and qualitative measures of physical activity and increased incidental exercise. Whilst this review aimed to assess studies where exposure to nature and not physical activity were the primary therapeutic mechanisms, studies did include some exercise to differing extents which evidence suggests can mediate the impact of NT on MI (Bélanger et al., 2019). For example, activities in Wilson et al. (2010) ranged in physicality from minimal (e.g., wreath making) to more physically demanding (e.g., building shelters out of branches). However, exposure to nature has impacts in its own right which are above and beyond the benefits of exercise (Pretty et al., 2007; Nisbet et al., 2009; Gladwell et al., 2013; Araújo et al., 2019; Brymer et al., 2020), and as such assessment of the relative impacts of exercise versus nature-exposure in these studies is important. While even small amounts of incidental exercise can improve wellbeing (Rosenbaum et al., 2014; Stubbs et al., 2018) individuals with MI are more likely to have physical illness that may prevent any exercise (Firth et al., 2019) knowing what nature-exposure interventions work without an exercise component is important. Within this review it is impossible to assess the extent of exercise in most included studies as the proportion of participants and or/time spent across these activities is unreported. Furthermore, even if this activity was reported it would be impossible to make a meaningful comparison across studies e.g., building bird feeders (Christie and Cole, 2017) versus bird watching (Maund et al., 2019). The heterogenous nature of NT and difficulty separating the impact of physical activity mean this generalizability and comparability will always be a limitation, however, detailed reporting in future research could somewhat address this problem. Additionally, careful consideration of the quantity and quality of incidental exercise is important for clinical practice, given the barriers to participation that this could present (Firth et al., 2016).

These findings suggest GSP can act as an effective form of diversional therapy for people with MI, providing opportunities for escape, leisure and socialization for participants, and reducing the impacts of loneliness and other social determinants of health. As there is some evidence that GSP may provide additional benefit where activities incorporate skill development and mastery, provide a routine, and facilitate social engagement, it would be of value for future research to evaluate the benefits of longer-term NT interventions with regular, ongoing sessions that incorporate these elements.

### Limitations

A limitation of this review is indicative of this area of the research in general in that many NT interventions have a physical activity component; this limits ability to identify the therapeutic impact of nature exposure alone and excludes potentially informative studies from this review. For example, a recent quantitative prospective study by Smyth et al. (2022) involved a SP “Green Gym” intervention with 892 participants with MI and/or long-term conditions, and showed significant improvements in participant wellbeing, with greater improvements for participants with poorer baseline wellbeing. This study used the WEMWBS (Stewart-Brown and Janmohamed, 2008) which is used in 2 included studies (Wilson et al., 2011; Maund et al., 2019), and included an appropriately long 13-month follow-up. Despite being a rigorous study in a peer-reviewed journal it does not meet the inclusion criteria for this review due to its primary focus on physical activity. However, this intervention included many activities that overlap with those in this review, e.g., gardening, weeding, and learning about local species (Smyth et al., 2022). It is also similar to that of Christie and Cole (2017) in which all activities are opt-in and range from learning about local species (very low physical activity requirements) to tree planting (high physical activity requirements). Smyth et al. (2022) and many papers in this review included incidental exercise, however, a greater focus on physical activity meant it did not strictly fit the inclusion criteria. Similarly Lindsay et al. (2022) conducted a novel SP fishing intervention in order to identify barriers to participation in GSP for people with long-term conditions. However, participant diagnoses were not collected or reported which precluded its inclusion in this review and also the conclusions that can be drawn about the efficacy of this novel GSP interventions for MI. This is both a limitation of this review and area of research.

An additional limitation of this review is that it only considered English-language literature; this may be a problem due to the emergence of types of NT particularly Forest Therapy in non-English-speaking countries such as Japan, China, and Korea. However, evidence suggests that excluding non-English studies generally does not impact the outcomes of reviews (Jüni et al., 2002).

## Recommendations

This review aimed to identify and critique extant GSP interventions in order to make recommendations for future research and clinical practice. These are as follows:

### Design and assessment

Stronger research designs including RCTs, improve reporting of qualitative assessments, and implement follow-up assessments. Clinicians should assess participant wellbeing pre- and post-intervention and consider publication to improve the evidence base.

### Recruitment

Reporting of referral pathways in detail and examine possible mediation effects. Clinicians should adapt the type and intensity of referral to individual client needs.

### Participant characteristics

Reporting of detailed demographic information and consider stratifying groups to explore demographics as mediators of effects. Clinicians should consider cohort demographics when referring to GSP programs to increase group cohesion.

### Setting

Reporting of environmental conditions in detail so a meaningful evidence base can be developed. Clinicians should consider local biome variability and match this to client needs.

### Intervention characteristics

Incorporation of skill development/sharing into GSP interventions. Clinicians should match this aspect of interventions to participant capacity and interests. Additionally, researchers should examine the benefits of both passive and active immersion in nature in particular mindfulness versus active engagement. Clinicians should consider client capacity when choosing GSP programs.

### Physical activity

Careful planning and reporting of the quality and quantity of physical activity within GSP interventions. Clinicians should consider physical activity capacity when referring clients.

## Conclusion

The primary finding of this review was that chronic under-reporting limits capacity to inform research and practice. In particular, intervention setting, participant characteristics, and recruitment strategies were poorly described, and without clarity on the “where, who, and how” it is difficult for these findings to be used to develop meaningful research and clinical practice recommendations. However, this review demonstrates that GSP can improve biopsychosocial wellbeing across a large variety of locations and intervention types. We conclude that future research in this growing area is worthwhile, but that detailed reporting of research methods is essential in order to develop a solid evidence base that can move the area forward. In terms of clinical practice, we conclude that GSP can improve biopsychosocial wellbeing in adults with MI, but unfortunately evidence is unclear on what works best for different groups. However, given the large variety in intervention types and activity levels we suggest that clinicians consider the needs and ability of the client before deciding which intervention to prescribe.

## Data availability statement

The original contributions presented in this study are included in the article/Supplementary material, further inquiries can be directed to the corresponding author.

## Author contributions

TT, CA, DM, and JB: conceptualization and methodology. TT, CA, DM, JB, MT, and DD’A: validation. TT, MT, and DD’A: formal analysis, investigation, and data curation. TT, CA, DM, JB, MT, DD’A, and EB: writing – original draft. TT, CA, JB, MT, DD’A, and EB: writing – review and editing. TT: visualization and project administration. CA, DM, JB, and EB: supervision. All authors contributed to the article and approved the submitted version.
